# Reduced complexity of activity patterns in patients with Chronic Fatigue Syndrome: a case control study

**DOI:** 10.1186/1751-0759-3-7

**Published:** 2009-06-02

**Authors:** Christopher Burton, Hans Knoop, Nikola Popovic, Michael Sharpe, Gijs Bleijenberg

**Affiliations:** 1 Division of Community Health Sciences, General Practice Section, University of Edinburgh, West Richmond Street, Edinburgh, UK; 2 Expert Centre Chronic Fatigue, Radboud University, Nijmegen, the Netherlands; 3 School of Mathematics and Maxwell Institute for Mathematical Sciences, University of Edinburgh, Kings Buildings, Edinburgh, UK; 4 Psychological Medicine Research, School of Molecular and Clinical Medicine, University of Edinburgh, Royal Edinburgh Hospital, Edinburgh, UK

## Abstract

**Background:**

Chronic fatigue syndrome (CFS) is an illness characterised by pervasive physical and mental fatigue without specific identified pathological changes. Many patients with CFS show reduced physical activity which, though quantifiable, has yielded little information to date. Nonlinear dynamic analysis of physiological data can be used to measure complexity in terms of dissimilarity within timescales and similarity across timescales. A reduction in these objective measures has been associated with disease and ageing. We aimed to test the hypothesis that activity patterns of patients with CFS would show reduced complexity compared to healthy controls.

**Methods:**

We analysed continuous activity data over 12 days from 42 patients with CFS and 21 matched healthy controls. We estimated complexity in two ways, measuring dissimilarity within timescales by calculating entropy after a symbolic dynamic transformation of the data and similarity across timescales by calculating the fractal dimension using allometric aggregation.

**Results:**

CFS cases showed reduced complexity compared to controls, as evidenced by reduced dissimilarity within timescales (mean (SD) Renyi_(3) _entropy 4.05 (0.21) vs. 4.30 (0.09), t = -6.6, p < 0.001) and reduced similarity across timescales (fractal dimension 1.19 (0.04) vs. 1.14 (0.04), t = 4.2, p < 0.001). This reduction in complexity persisted after adjustment for total activity.

**Conclusion:**

Patients with CFS show evidence of reduced complexity of activity patterns. Measures of complexity applied to activity have potential value as objective indicators for CFS.

## Background

Chronic Fatigue Syndrome (CFS) is a disorder characterised by profound physical and mental fatigue, with additional characteristic symptoms [1,2]. While the exact cause remains unknown, both physiological and psychological processes have been implicated [3-5]. Although many of the symptoms of CFS are experienced within the body, increasing evidence points to altered central processing of sensations of fatigue and physical activity [4]. Continuous measurement of physical activity (actigraphy) indicates that while some CFS patients show markedly reduced activity, others cannot be distinguished from healthy controls using conventional statistical techniques [2,6]. The underlying physiological mechanisms which drive activity and rest are not fully understood, but recent work has identified a coherent structure to the distribution of long and short periods of activity, leading to the suggestion that the qualitative pattern of spontaneous activity over time may be centrally determined [7].

The analysis of complex patterns over time has been carried out for a number of physiological processes including heart rate variability [8], respiratory function [9] and gait [10] using techniques derived from the physics of nonlinear dynamic systems. Typically, this data shows two features: short-range (or within-scale) dissimilarity and long-range (or across-scale) similarity. (Here, scale refers to the choice of a particular viewing frame, typically with reference to space or time.) Together, these features are regarded as quantifiable characteristics of complexity which can be observed both in structure (complexity in space) and in behaviour (complexity in time). Work over the last 15 years has repeatedly demonstrated that health is a state of high complexity compared to disease and ageing [11]. At first sight this appears paradoxical: the traditional view of healthy homeostasis is of relative constancy and order, which is then perturbed by the "disorder" of disease; however, as Goldberger and others have pointed out [11,12], high complexity implies constant adaptability to an ever-changing environment, whereas low complexity implies inflexibility as is seen, for instance, in the unvarying stride length of patients with Parkinson's Disease [12]. An example of the concept of within-scale dissimilarity and across-scale similarity is given below.

Two key features of complexity, dissimilarity within the same scale and similarity across scales ("self-similarity"), can be seen in the simple example of the structure of a tree and the way it branches. When viewed from the same distance, each branch of about the same size is different from every other in its branching pattern. However, when viewed from different distances, by "zooming" from far away – whereby only the trunk and main branches are seen – to close up – so that only a few outermost branches are visible – the branching pattern appears similar.

A measure of complexity in this case might thus include two components: how different each branch is compared to other branches of the same size, and how similar in pattern (but not size) branches of different size are.

Applying this approach to time series data, one would ask how different the activity is in each short period compared to other similar periods (dissimilarity within the same timescale), and how similar the stop and start patterns seen over 15 minutes are to those observed over 2 hours or over 12 hours (similarity across timescales).

Because conventional analysis of actigraphic data is unable to differentiate healthy controls from some, but not all, patients with CFS [6], and because of the suggestion that activity patterns represent the output of a complex central system [7], we sought to apply nonlinear dynamic analysis to actigraphy data from patients with CFS. To date, there has only been one published application of nonlinear dynamics to actigraphy data in CFS patients. That study found less similarity across timescales in cases than controls [13], in keeping with the hypothesis that illness is associated with loss of complexity, but did not include a measure of dissimilarity within timescales.

### Aims

We aimed to test the hypothesis that objective measures of movement in both active and inactive patients with CFS would show reduced complexity – defined as dissimilarity within timescales and similarity across timescales – compared to healthy controls.

## Methods

### Data collection

We used data from previous studies [6] of spontaneous movement in CFS in which data had been collected using a solid state activity meter (Actilog V3.0) worn continuously on the ankle. Data was recorded continuously over 12 days and saved as counts of movements for each five-minute period. No new data was collected for the purposes of this study. Participants had given informed consent for the recording and subsequent analysis of data through the ethical committee of the local institution.

We identified recordings from three categories of individuals defined in an earlier study [6] on the basis of clinical assessment and the overall pattern of the activity recordings: healthy controls, CFS patients with relatively normal activity (active CFS) and CFS patients with clearly reduced activity (inactive CFS). All cases met CDC criteria for CFS [1]. Actigraph recorders were fitted to participants' ankles – giving them the choice for comfort – to avoid issues of dominance which would occur if the devices were applied to the wrist. Recordings were available from 21 healthy controls, so we selected the nearest match in age and sex from each of the two CFS groups. Although our earlier work [6] had suggested three categories of activity: pervasively inactive, relatively active and pervasively active, in accordance with our current clinical practice the relatively and pervasively active patients were grouped together.

### Statistical Methods

Simple descriptive analysis of the data included mean, standard deviation and proportion of five-minute periods in which no movement was recorded. Measures of dissimilarity within timescales (entropy) and similarity across timescales (fractal dimension) were calculated for each individual participant, using methods described below, and results were compared between groups. Nonlinear dynamic measures were calculated using specifically written scripts in R, an open source language and environment for statistical computing and graphics [14], which was also used for the subsequent comparisons between cases and controls.

#### Dissimilarity within timescales

For the analysis of dissimilarity within timescales, we calculated informational entropy using symbolic dynamics [15]. Entropy measures [11] can be used to describe the degree of variability in a data series and are derived from the notion that the more particular patterns recur in the data, the less information is required to describe the whole series. Thus, if one measures the frequency distribution of short subsequences within a larger data series, a low-entropy series will contain more recurring subsequences than one with high entropy.

We used the technique of symbolic dynamics to prepare the data for analysis. This approach converts a sequence of continuous values to discrete "words" representing subsequences in three steps. First, we condensed the individual time series to a limited number of values, in this case using terciles. Second, we represented each point as a symbol and third, we reconstructed the series as a sequence of overlapping qualitative "words" of a given number of symbols: for this study, we used a word length of 3 symbols. As an example, a sequence of values 1,8,7,10,1,3,5,12,7 would be converted to terciles (1,3,2,3,1,1,2,3,2), which were then translated to symbols (a, c, b, c, a, a, b, c, b). Finally, the sequence of symbols would be converted to overlapping three-letter words ("acb","cbc","bca",...).

Considering the words generated by this method, it can be understood that a completely random numerical series should generate all words with equal probability, whereas a simple periodic system (for instance 2,3,2,3,2,3,...) will generate only a small number of words – in this case two, "bcb" and "cbc".

A number of measures have been proposed to quantify the dissimilarity in an information series: two such established measures are Shannon entropy and Renyi entropy, and these have previously been used in the analysis of the symbolic dynamics of heart rate variability [15]. Shannon entropy is calculated for a set X of n individual words (x_1_, x_2_, x_3_,..., x_n_) using the probability p(x_i_) of each word x_i _occurring, as shown in Equation 1.

(1)

For a random series of words comprising three symbols each with three possible values, the value of Shannon Entropy is 4.75.

Renyi entropy (Equation 2) represents a generalisation of the special case of Shannon entropy to include an additional variable q which has the effect of increasing the dependence of the statistic on words which either occur frequently (q > 1) or infrequently (q between 0 and 1) [15]. For this study, we used the values for q of 3 and 0.33 used previously in the analysis of heart rate variability in [15].

(2)

To test the sensitivity of the algorithm to finite-size data series, we calculated Shannon and Renyi entropy for 50 series comprising 1200 uniformly distributed random numbers. The mean (SD) value for Shannon Entropy was 4.73 (0.006), close to the theoretical value of 4.75, and for Renyi Entropy 4.71 (0.018), with the parameter q = 3 (hereafter referred to as Renyi_(3)_).

After preliminary analysis of the data, we identified three necessary additional steps of data preparation. First, we removed all periods of prolonged rest, as the number of these differed between groups and we specifically wished to investigate activity. Rest was defined as three consecutive five-minute periods in which the mean activity count was below a given proportion of the mean activity count of the whole series. We carried out the analysis using thresholds of 16%, 25% and 33% of the mean series value. These were chosen arbitrarily after visual inspection of the data in order to provide a reasonably unbroken period of rest overnight while not obscuring brief periods of rest or reduced activity by day. Second, we differenced the data so that the series represented changes in activity rather than absolute activity levels; thus, the three symbols we used representing terciles of the individual differenced series can be interpreted as "reduce activity", "keep to around the same level of activity" and "increase activity". Cut points between the terciles were applied to each series individually rather than using pooled data. Third, we truncated all series to the length (after removal of resting periods) of the shortest.

#### Similarity across timescales

We measured similarity across timescales by allometric aggregation [16], which was developed by West [12] and colleagues to characterise scaling in biomedical data series and which can be used to derive the fractal dimension of the underlying process.

Allometric aggregation exploits the relationship between the mean and variance of a data series as the data points are iteratively aggregated into subseries. For a sequence of length L and a range of values S (ranging from 1 to approximately L/10), the sequence is divided into L/S subsequences, or blocks. The value of each block is determined as the sum of its component points, and at each block size the mean and variance for the set of blocks is calculated. As block size increases, the relationship between mean and variance stays fixed and can be described as a linear relationship between log(variance) and log(mean). The coefficient β of this relationship is related to the local Hölder exponent h by the equation β = 2 h, and thus to the fractal dimension D, as shown in Equation 3 [16]. Consequently, D decreases with increasing β or, equivalently, with increasing h. In the allometric aggregation of the data, we considered all data points, including those with zero activity, when calculating the fractal dimension.

(3)

Our analysis of across-scale similarity was based on identifying correlations across multiple scales (so-called fractal correlations) in the data. Where those correlations are high, patterns are similar when viewed at different scales; conversely, they are dissimilar where correlations are low. The correlation strength increases with the local Hölder exponent h or, equivalently, with the fractal scaling exponent β = 2 h obtained from allometric aggregation [16]. Alternatively, the fractal dimension D can be taken as a measure of the information required to describe a system across different scales. By Equation 3, D is negatively related to the fractal correlation, so that, as correlation increases (towards regularity), the fractal dimension decreases. Likewise, as correlation decreases (towards randomness), the dimension increases. In the context of a unidimensional time series, D = 1 corresponds to a completely regular process, whereas D = 1.5 represents an uncorrelated random process; healthy physiological processes operate somewhere in between these two extremes [12].

#### Comparison of measures of complexity between individuals

We used t-tests to compare the measures of dissimilarity within timescales (Shannon and Renyi entropy) in order to select the statistic that appeared to differentiate best between the controls and the two CFS groups. We then used analysis of covariance to identify the effect of group on the best-performing entropy statistic and on fractal dimension after adjusting for average activity. Separate comparisons were made for the active and inactive CFS cases with controls using dummy variables (control: 0 and case: 1). Finally we carried out a sensitivity analysis in which the two patients taking antidepressants or patients originally rated as pervasively active (2 active CFS cases and 8 controls) were removed from the analysis.

## Results

We report data from 63 participants: 21 normal controls and 42 CFS cases equally divided between the active CFS and inactive CFS groups. Baseline characteristics are shown in Table 1.

**Table 1:** Patient characteristics and summary actigraphy results by participant group

	**CFS inactive**		**CFS active**		**Controls**	
	
Number	21		21		21	
Age	37.4		38.0		34.8	
Female (%)	16	(76%)	16	(76%)	16	(76%)
Mean actigraphy score (SD)	26	(7.0)***	50	(10.0)*	60	(17.0)
Mean number of periods with zero movement (%)	1765	(51%)***	1466	(42%)**	1237	(36%)

t-test comparison with normal controls: *p < 0.05 ** p < 0.01 *** p < 0.001

Total periods for each individual: 3456.

### Dissimilarity within timescales (entropy)

**Figure 1:**
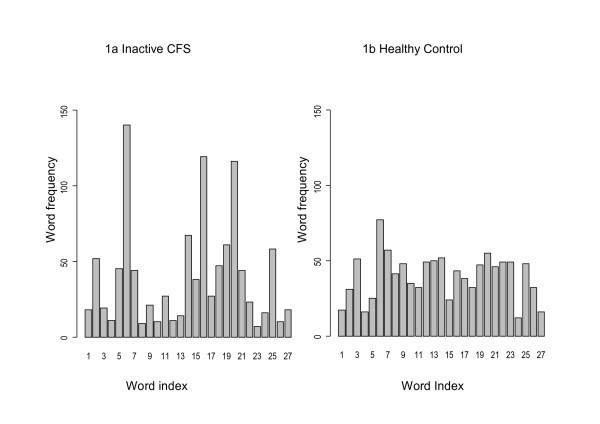
**Histograms of the "word" distribution after symbolic dynamic transformation of one inactive CFS case and one healthy control**. Word index refers to the sequence of 3 letter "words" where 1= "000", 2="001" ... 27="222"

Figure (1a and 1b) shows two examples of the word distribution, one an inactive CFS case (Renyi_(3) _entropy 3.71) and one a healthy control (Renyi_(3) _entropy 4.50). The histogram in panel (a) shows a concentration on a small number of patterns, whereas the histogram in panel (b) is characterised by a more even distribution with smaller peaks.

Measures of entropy showed no relation to age (Renyi_(3) _correlation coefficient r = 0.08, p = 0.5) or sex (mean (SD) Renyi_(3) _4.13 (0.2) and 4.12 (0.2) for males and females, respectively). Table 2 shows the mean and standard deviations for normal controls and the two categories of cases for Renyi_(3)_, Renyi_(0.33) _and Shannon entropy. From these, we selected Renyi_(3) _entropy as the statistic offering the best discrimination between groups. Renyi_(3) _entropy was reduced in CFS cases (pooled active and inactive) compared to controls: mean (SD) 4.05 (0.21) vs. 4.30 (0.09), t = -6.6, p < 0.001.

**Table 2:** Comparison of complexity measures by group

	**Controls**		**Active CFS**			**Inactive CFS**		
	
	mean	SD	mean	SD	t, p-value	Mean	SD	t, p-value
	
Renyi(3) Entropy	4.30	0.09	4.17	0.15	3.4 p = 0.002	3.93	0.19	7.9 p < 0.001
Renyi(0.33) Entropy	4.69	0.01	4.67	0.02	2.1 p = 0.05	4.64	0.03	6.9 p < 0.001
Shannon Entropy	4.56	0.04	4.52	0.07	2.5 p = 0.02	4.41	0.09	7.4 p < 0.001
								
Fractal Dimension	1.14	0.04	1.16	0.02	1.8 p = 0.07	1.21	0.03	6.3 p < 0.001

t-tests and corresponding p values are compared to healthy controls

Results of the analysis of covariance, including average activity scores, are shown in Table 3. These show that after adjusting for average activity, the coefficient for category remained significant for both active CFS (β = -0.07, p = 0.027) and inactive CFS (β = -0.18, p = 0.009) when Renyi_(3) _was the dependent variable.

**Table 3:** Regression coefficients for diagnostic category with complexity measures as dependent variables

**Measure**		**Active CFS**		**Inactive CFS**	
	Covariate	β	p^2^	β	p^3^
			
Renyi(3) Entropy					
	none	-0.13	0.002	-0.37	<0.001
	Mean activity^4^	-0.07	0.027	-0.18	0.009
Fractal Dimension	
	none	0.02	0.08	0.066	<0.001
	Mean activity^4^	0.004	0.6	0.03	0.05

Active CFS and Inactive CFS groups analysed separately.

^2^Active CFS (N = 21) vs. Control (N = 21).

^3^Inactive CFS (N = 21) vs. Control (N = 21).

^4 ^Mean number of movements per block.

The entropy calculations were repeated excluding data based on rest periods of 16% and 33% of the mean activity per five-minute period and with word lengths of 4 symbols. The results for Renyi_(3) _entropy are shown in Table 4. When the entropy calculations were repeated with undifferenced data, we found no significant difference between cases and controls. Repeating the entropy calculations after randomly shuffling each series to break up the structure of the time series also removed the difference between cases and controls.

**Table 4:** Comparison of Renyi(3) entropy obtained using different activity thresholds and word length

**Parameter**		**Controls**		**Active CFS**		**Inactive CFS**	
Word length^1^	Activity threshold^2^	Mean	SD	Mean	SD	Mean	SD
		
3	0.25	4.30	0.09	4.18	0.15	3.93	0.20
3	0.33	4.33	0.09	4.20	0.17	3.92	0.22
3	0.16	4.30	0.08	4.21	0.10	3.99	0.19
4	0.25	5.58	0.12	5.39	0.22	5.04	0.31

^1^Word Length: number of symbols comprising a "word" for symbolic dynamic analysis.

^2^Activity threshold: proportion of individual participants' mean activity below which five minute blocks were excluded from symbolic dynamic analysis.

### Similarity across timescales (fractal dimension)

**Figure 2:**
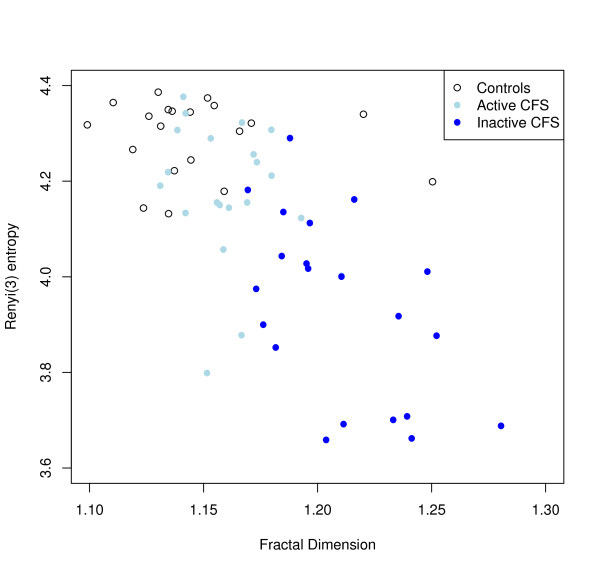
**Scatter plot of dissimilarity within scales (Renyi entropy) and similarity across scales (fractal dimension)**.

The fractal dimension D derived from the coefficient of the allometric aggregation relationship between log(mean) and log(variance) was lower for normal controls than for pooled CFS cases: mean(SD) 1.14 (0.04) and 1.19 (0.04), respectively (t = 4.2, p < 0.001), indicating a loss of similarity across timescales in CFS cases. Table 2 shows the mean and standard deviation for D in controls and each of the two patient categories. The results of the analysis of covariance are given in Table 3. There was a significant correlation between Renyi entropy and fractal dimension from allometric aggregation (r = -0.68, p < 0.001); a scatter plot illustrating the relationship between these two measures is shown in Figure 2.

### Sensitivity analysis

When the patients on antidepressants were excluded there was no change in regression coefficients. When the 10 pervasively active patients were excluded the regression coefficient for active CFS compared to controls on Renyi_(3) _entropy, adjusting for mean activity, was unchanged although the p value was increased reflecting the reduced sample size (β = -0.07, p = 0.067).

## Discussion

### Summary of main findings

This is the first study to report reduced complexity both within timescales and across timescales in the activity patterns of patients with CFS. Differences to healthy controls were seen in both active and inactive CFS cases. Regarding loss of dissimilarity within timescales, the differences in entropy measures between cases and controls remained statistically significant after adjusting for other characteristics of the data. Reduced similarity across timescales was accompanied by an increase in fractal dimension or, alternatively, by a reduced fractal scaling exponent, in keeping with previous studies of complexity loss through ageing and neurodegenerative disease [12]. These findings support our hypothesis that CFS cases would show greater evidence of reduced complexity than controls and are in keeping with the model of disease as loss of complexity [11]. They also suggest a new and quantifiable feature of CFS.

### Strengths and weaknesses of the study

The data used for this study came from continuous recordings made during normal daily activities with few sources of bias in collection. Only objective data from the activity meters was used. All cases met current research diagnostic criteria for CFS, and the study included groups of patients with different severity as judged by total amount of activity. The methods of analysis have both been described elsewhere [15,16] and shown to have discriminatory power in different contexts. Unlike some nonlinear analyses, they do not assume the presence of mathematical phenomena such as deterministic chaos [17], nor do they require very large sample sizes.

However, a number of arbitrary steps were introduced into the methods, and their role needs to be considered. The decision to eliminate rest periods for the analysis of dissimilarity within timescales was necessary because regularity statistics such as measures of entropy identify repeating patterns; if rest periods were not excluded, then sequences of zeros would become the dominant feature in the data. We considered only allowing recordings between certain times of day, but found that cases and controls differed in the timing and duration of apparent night-time and daytime rest periods and that only eliminating data with absolute zeros did not exclude minor night-time movements. Our method of allocating periods to activity or rest according to a threshold is in keeping with the approach taken by others [7].

Although Voss [18], who carried out a similar analysis with heartbeat series of 1200 points, used four- letter "words", we chose three letters, to represent increased activity, reduced activity and approximately unchanged activity. The decision to use differenced data reflecting changes in activity rather than absolute activity levels was made after inspecting the distribution of the undifferenced data and finding that cut points between terciles were relatively close together at low activity levels when undifferenced data was used.

### Comparison with other studies

Most studies of dissimilarity within timescales in other conditions have used quantitative measures such as approximate entropy [19], or the related sample entropy [8], and their findings have generally supported the association between disease and loss of complexity. However, these methods depend on data which is distributed relatively uniformly around a mean value, a feature that the heavily skewed data used here did not possess. Techniques involving symbolic dynamics, which are less dependent on the fine-grained characteristics of the data series, have been applied successfully to address similar questions [15,20]. Similarity across scales and long-term correlations in data have been addressed using a range of measures, typically detrended fluctuation analysis [21]. However, like Ohashi et al. [13], we found that this did not differentiate cases from controls (data not shown). In their study, they measured similarity across timescales using the negative modulus maxima of the wavelet-transformed activity data which specifically relates to the cessation of activity. The results of our study were reported in terms of fractal correlations, which are negatively related to the fractal dimension: hence, the smaller correlations in CFS cases found by Ohashi et al. [13] are equivalent to the higher fractal dimension reported here; however, their study was smaller, and was unable to differentiate active CFS cases from controls.

Recently, the organisation of physical activity patterns, whereby periods of activity and rest are interwoven, has been shown to have a coherent structure across individuals and across timescales from 2 to 200 minutes [7,22]. The activity patterns which we report are compatible with the complex physiological control system proposed to explain this finding.

### Implications for future research and practice

Further study should investigate whether the reduced complexity we found is reversed in patients who recover, and how it relates to the changes in endocrine [3], immunological and cognitive function which are also seen in some patients with CFS. The notion that spontaneous activity patterns are centrally and involuntarily organised [7], and that the characteristics of this organisation are changed in CFS, may also provide useful foundations for explanatory models for this condition, in which rival explanations are frequently contested between patients and their doctors.

## Conclusion

The spontaneous activity patterns of patients with CFS show significantly reduced complexity, compared to normal controls. This finding is similar to the reduced complexity seen in several other conditions and has potential as an objective marker for CFS which merits further investigation.

## Abbreviations

CFS: Chronic Fatigue Syndrome; SD: Standard Deviation.

## Competing interests

The authors declare that they have no competing interests.

## Authors' contributions

CB conceived of the study and carried out the analysis of entropy and fractal dimension. HK and GJ carried out the original clinical study and informed the analysis reported here. NP and MS provided advice on the study design and assisted in drafting the manuscript. All authors read and approved the final manuscript.
